# Supplementary material for: Digital health interventions in strengthening primary healthcare systems in Sub-Saharan Africa: Insights from Ethiopia, Ghana, and Zimbabwe
Source: PLOS Digit Health. 2026 Jan 2;5(1):e0000863. doi: 10.1371/journal.pdig.0000863 (PMC12758792; doi:10.1371/journal.pdig.0000863)
Supplement: Attachment — Submitted filename: Tracked Revisions Updated Nov .pdf [file pdig.0000863.s003.pdf]

Table summarising the requested changes

Reviewer	Request	Response
**Journal Requirements:**	**Nov Requests**<br><br>1. Please amend your detailed Financial Disclosure statement. This is published with the article. It must therefore be completed in full sentences and contain the exact wording you wish to be published.<br><br>i.. State the initials, alongside each funding source, of each author to receive each grant.<br>ii. State what role the funders took in the study. If the funders had no role in your study, please state: "The funders had no role in study design, data collection and analysis, decision to publish, or preparation of the manuscript."<br><br>2. Please ensure that the funders and grant numbers match between the Financial Disclosure field and the Funding Information tab in your submission form. Note that the funders must be provided in the same order in both places as well.<br><br>3. Thank you for uploading your study's underlying data set. Unfortunately, the repository you have noted in your Data Availability statement	**Financial Disclosure Statement**<br><br>This work received financial support from the WHO Alliance for Health Policy and Systems Research (Alliance) Zimbabwe (Grant Numbers 2023/1325412 and 2024/1440425), Ghana (Grant Numbers 2023/1330956 and 2024/1442222, and Ethiopia (Grant Numbers 2023/1326656 and 2024/1441908). The Alliance is able to conduct its work thanks to the commitment and support from a variety of funders. These include our long-term support from the Swedish International Development Cooperation Agency (SIDA) and the Norwegian Agency for Development Cooperation (NORAD), as well as designated funding for specific projects within our current priorities. For the full list of Alliance donors, please visit https://ahpsr.who.int/aboutus/ funders.<br><br>The funders had no role in the study design or data collection and analysis, preparation of the manuscript, or the decision to submit it for publication. All authors declare that they have no other financial relationships that could be perceived to influence the work reported in this paper.<br><br>**Response:**<br><br>All the funding information required has been harmonised between the submission for and funding information tab<br><br>**Response:**

	does not qualify as an acceptable data repository according to PLOS's standards.<br><br>At this time, please upload the minimal data set necessary to replicate your study's findings to a stable, public repository (such as figshare or Dryad) and provide us with the relevant URLs, DOIs, or accession numbers that may be used to access these data. For a list of recommended repositories and additional information on PLOS standards for data deposition, please see https://journals.plos.org/digitalhealth/s/recommended-repositories.<br><br>4. Please ensure that the Title in your manuscript and the Title in your online submission form are the same.<br><br>5. Please ensure that the Author List in your manuscript file matches the Author List in the online submission form exactly. Authors should be listed in the same order, and the order of individual first and last author names must be identical in both locations.	This has been addressed in **Figshare**<br><br>DOI for the study:'<br><br>10.6084/m9.figshare.30646865<br><br>**Response:**<br><br>This is addressed. The title is<br><br>" Digital Health Interventions in Strengthening Primary Healthcare Systems in Sub-Saharan Africa: Insights from Ethiopia, Ghana, and Zimbabwe"<br><br>**Response:**<br><br>This is addressed. The Author List is as follows:<br><br>Simbini T, Adimado E[2], Adjorlolo S, Guerrero-Torres L[5], Srinivas P N[6], Zizhou S[7], Zerfu TA[8]

	**Oct Feedback**<br><br>1. Please amend your detailed Financial Disclosure statement. This is published with the article. It must therefore be completed in full sentences and contain the exact wording you wish to be published.<br><br>i.. State the initials, alongside each funding source, of each author to receive each grant.<br>ii. State what role the funders took in the study. If the funders had no role in your study, please state: "The funders had no role in study design, data collection and analysis, decision to publish, or preparation of the manuscript."	The financial disclosure statement have been revised accordingly. The revised statement reads:<br><br>**Financial Disclosure Statement**<br><br>This work was supported by country specific grants from the **WHO Alliance for Health Policy and Systems Research (Alliance)** to the participating researchers in Zimbabwe (Simbini T, Zizhou S), Ghana (Adimado E, Adjorlolo S), and Ethiopia (Zerfu T A). The funders, **Guerrero-Torres L** and **Srinivas P N**, contributed to the decision to publish and to the review of the manuscript, but had no role in the study design or data collection. The funders had no additional role in data analysis, preparation of the manuscript, or the decision to submit it for publication. All authors declare that they have no other financial relationships that could be perceived to influence the work reported in this paper.

	2. Please ensure that your Ethics Statement is available in its entirety at the beginning of your Methods section, under a subheading 'Ethics Statement'.	**Response:** We have moved the sections around. Ethics Statement and Ethical Approvals have been moved and sub-headed Methods section.
	We ask that a manuscript source file is provided at Revision. Please upload your manuscript file as a .doc, .docx, .rtf or .tex.	Two files in .docx are provided one with extension "...track changes.docx" and the other "..clean final"<br><br>This is done. However due to the persistent revisions of the paper we were not able to track all the changes. The clean final is the main paper.
	4. In the online submission form, you indicated that "The data is largely qualitative interviews in Excel Sheets. These can be made available online as requested".<br><br>All PLOS journals now require all data underlying the findings described in their manuscript to be freely available to other researchers, either<br>1. In a public repository,<br>2. Within the manuscript itself, or<br>3. Uploaded as supplementary information.	We have provided the data sources in a google drive folder below"<br><br>https://drive.google.com/drive/folders/1h0wLSF-i2shYn47xb9onCnxW5sAWnUSY?usp=sharing
Reviewers	3. Have the authors made all data underlying the findings in their manuscript fully available (please refer to the Data Availability Statement at the start of the manuscript PDF file)?<br><br>The PLOS Data policy requires authors to make all data underlying the findings described in their manuscript fully available without restriction, with	This data is available in this folder.<br><br>https://drive.google.com/drive/folders/1h0wLSF-i2shYn47xb9onCnxW5sAWnUSY?usp=sharing

	rare exception. The data should be provided as part of the manuscript or its supporting information, or deposited to a public repository. For example, in addition to summary statistics, the data points behind means, medians and variance measures should be available. If there are restrictions on publicly sharing data—e.g. participant privacy or use of data from a third party—those must be specified.<br><br>Reviewer #1: Yes<br>Reviewer #2: No<br>Reviewer #3: No	
Reviewer 1	Reviewer #1: The manuscript is essential in its provision of a relevant analysis of Digital Health Interventions in three African countries. It offers valuable insights into their role in strengthening health systems. The study has an excellent structure with good methodology while addressing critical gaps in literature. Please find a few comments below for your consideration.<br><br>Sampling Details: Provide more information on the sampling strategies (e.g., criteria for selecting regions/facilities in each country) to enhance reproducibility.<br><br>Data Analysis: Clarify how qualitative data (e.g., interviews, FGDs) were analyzed (e.g., software used, coding framework). For quantitative data, consider including statistical measures (e.g., p-values, confidence intervals) where applicable.<br><br>Policy Recommendations: Strengthen the conclusion by explicitly linking findings to	We have added the following actionable recommendations at the end of the conclusion<br>"Below are actionable recommendations for policymakers, funders, and implementers:<br>**Policymakers** should prioritize strengthening all health system building blocks while integrating digital health interventions (DHIs). DHIs should be viewed as enablers of health systems strengthening rather than as standalone or parallel implementations.<br>**Funders** should support DHIs as comprehensive programs that emphasize systems integration and alignment within the broader health system. Investments should promote sustainable ecosystems of interoperable solutions, underpinned by strong human resource capacity and long-term operational support.<br>**Implementers** should ensure interoperability across platforms, address infrastructure limitations to guarantee the availability of mission-critical DHIs, and optimize user interfaces and user experiences (UI/UX) for seamless data entry at the point of care. Additionally, robust change management strategies should be embedded to support user adoption and system sustainability."<br><br>Sampling details we have added:<br>"The team used purposive convenient sampling selecting all secondary healthcare facilities located in the Greater Accra region that uses both the DHIMS and LHIMS. For the study participants, purposive sampling of 125 primary healthcare managers from the healthcare facilities who were familiar and ready to provide information on the DHIMS and LHIMS."

	actionable recommendations for policymakers, funders, and implementers.	For Statistical Analysis we have stated:<br>Ghana "Quantitative data were analyzed using descriptive statistics in STATA. For the qualitative component, transcripts from KIIs and in-depth interviews were manually coded using an inductive thematic analysis approach, drawing on established frameworks such as Braun and Clarke's six-step mode"<br>Zimbabwe "Qualitative data were collected through audio recordings and manually transcribed into Microsoft Excel® sheets, organized by question and response. Each question was reviewed and summarised to identify common themes, which were further refined using a large language model for thematic synthesis and summary generation. Quantitative descriptive statistics, primarily frequencies, were computed in Excel®"
Reviewer 2	Reviewer #2: There are several ambiguities across the manuscript which needs to be solved before sending for peer reviewing. The structure and design of the article is unclear. It is not clear why three separate studies from three countries were combined and presented in the form of one manuscript. Normally, if the reader is interested in the topic, he/she will refer to the three original articles. *Why were the three countries chosen and based on what criteria was this choice made?* Detailed comments have been included in the attached file.	**Response:**<br><br>This sentence was added at the end of the introduction<br><br>"*These countries received country-specific grants from the WHO Alliance for Health Policy and Systems Research (the Alliance) to conduct studies on digital health interventions (DHIs), each implemented independently. This article synthesizes and consolidates common themes emerging across the three studies"*<br><br>We appreciate the reviewer's comments in the attached file. We acknowledge that our initial submission did not clearly specify that this article synthesizes findings from three separate country studies conducted by independent research teams. We have now clarified this point by explicitly stating that the article consolidates and summarizes the results from these country-specific studies.<br><br>The section titled *"DHIs Implementation Context"* is intended to provide readers with background information on the maturity level of DHI implementation in each country. For this reason, we have retained it in the background section rather than moving it to the Results, as it provides essential contextual information rather than study findings.
Reviewer 3	Reviewer #3: - Underlying data isn't available yet, although the authors indicated that it can be made	Response:<br><br>Has been provided on the google drive link

	available as requested.<br>- The methods section is fair enough, however, it would help to include more details of specific tools/software used for analyses. Also, for the Ethiopia study, the exact number of key informant interview and focus group discussion participants across the 5 regions were not indicated.<br><br>To the authors: Highly relevant study, however it could benefit from more evidence support. For instance, in the strategies recommended in the Discussion section, it would help to include references to studies that show how these strategies have worked in similar contexts.	Discussion: We have revised the entire Discussion section to take into consideration the raised points here.<br><br>Targeted Training & Mentorship<br>References have been provided. Ref 12 to 19)

We have revised the Results section to include the Governance and Leadership track of the health systems building blocks.

Governance and Leadership

The findings from the three studies note the increased accountability by decision makers at each administrative level, hence strengthening health systems governance structures. In Ethiopia there is 96% usage of DHIS2 for program monitoring, and 87% for resource allocation at primary health units, increasing the availability of programme data strengthening decision making and accountability at an administrative level. In Ghana, the researchers have reported an increase in accountability using the DHIMS system, with 70% of the primary care managers obtaining summary data from DHIMS. In Zimbabwe, the availability of data in the medicines supply chain to nurses in the frontline has demonstrated the increased accountability of health services. Hence these findings infer on the DHIs impact on this building block.

Discussion

We have revamped the discussion section to summarize the findings that speak towards specific strategies for different audiences.

We have also added limitations to the paper as shown below.

Limitations

The purpose of combining these three independent studies was to identify common themes and cross-cutting opportunities across different country contexts. However, several limitations should be noted. Each study was conducted by separate research teams, using distinct methodologies, analytical frameworks, and timelines. As a result, direct comparisons between the studies may be constrained by variations in study design, data collection tools, and contextual factors. Furthermore, while efforts were made to synthesize the findings objectively, the integration of independently generated data introduces a degree of interpretive bias. Despite these limitations, the synthesis provides valuable insights into shared experiences and lessons for digital health implementation in the three African country contexts. .
